# Chemical exchange saturation transfer MRI serves as predictor of early progression in glioblastoma patients

**DOI:** 10.18632/oncotarget.25594

**Published:** 2018-06-19

**Authors:** Sebastian Regnery, Sebastian Adeberg, Constantin Dreher, Johanna Oberhollenzer, Jan-Eric Meissner, Steffen Goerke, Johannes Windschuh, Katerina Deike-Hofmann, Sebastian Bickelhaupt, Moritz Zaiss, Alexander Radbruch, Martin Bendszus, Wolfgang Wick, Andreas Unterberg, Stefan Rieken, Jürgen Debus, Peter Bachert, Mark Ladd, Heinz-Peter Schlemmer, Daniel Paech

**Affiliations:** ^1^ Department of Radiation Oncology, University Hospital Heidelberg, Heidelberg, Germany; ^2^ German Cancer Research Center (DKFZ), Division of Radiology, Heidelberg, Germany; ^3^ German Cancer Research Center (DKFZ), HIRO (Heidelberg Institute for Radiation Oncology), Heidelberg, Germany; ^4^ German Cancer Research Center (DKFZ), Division of Medical Physics in Radiology, Heidelberg, Germany; ^5^ Max-Planck-Institute, Tübingen, Germany; ^6^ Department of Neuroradiology, University Hospital Heidelberg, Heidelberg, Germany; ^7^ Department of Neurology, University Hospital Heidelberg, Heidelberg, Germany; ^8^ Department of Neurosurgery, University Hospital Heidelberg, Heidelberg, Germany; ^9^ Faculty of Physics and Astronomy, University of Heidelberg, Heidelberg, Germany; ^10^ Faculty of Medicine, University of Heidelberg, Heidelberg, Germany

**Keywords:** magnetic resonance imaging, amide-proton-transfer-imaging, nuclear overhauser imaging, glioblastoma, predictive biomarker

## Abstract

**Purpose:**

To prospectively investigate chemical exchange saturation transfer (CEST) MRI in glioblastoma patients as predictor of early tumor progression after first-line treatment.

**Experimental Design:**

Twenty previously untreated glioblastoma patients underwent CEST MRI employing a 7T whole-body scanner. Nuclear Overhauser effect (NOE) as well as amide proton transfer (APT) CEST signals were isolated using Lorentzian difference (LD) analysis and relaxation compensated by the apparent exchange-dependent relaxation rate (AREX) evaluation. Additionally, NOE-weighted asymmetric magnetic transfer ratio (MTRasym) and downfield-NOE-suppressed APT (dns-APT) were calculated. Patient response to consecutive treatment was determined according to the RANO criteria. Mean signal intensities of each contrast in the whole tumor area were compared between early-progressive and stable disease.

**Results:**

Pre-treatment tumor signal intensity differed significantly regarding responsiveness to first-line therapy in NOE-LD (*p* = 0.0001), NOE-weighted MTRasym (*p* = 0.0186) and dns-APT (*p* = 0.0328) contrasts. Hence, significant prediction of early progression was possible employing NOE-LD (AUC = 0.98, *p* = 0.0005), NOE-weighted MTRasym (AUC = 0.83, *p* = 0.0166) and dns-APT (AUC = 0.80, *p* = 0.0318). The NOE-LD provided the highest sensitivity (91%) and specificity (100%).

**Conclusions:**

CEST derived contrasts, particularly NOE-weighted imaging and dns-APT, yielded significant predictors of early progression after fist-line therapy in glioblastoma. Therefore, CEST MRI might be considered as non-invasive tool for customization of treatment in the future.

## INTRODUCTION

Glioblastoma patients face a dismal prognosis despite optimal standard treatment consisting of resection followed by adjuvant chemoradiotherapy (CRT) [1–4]. However, a small group survives considerably longer [1, 3]. Hence, biomarkers predicting tumor response towards first-line treatment are highly desirable. Currently, histopathological analysis of O6-methylguanine DNA methyltransferase (MGMT) promotor methylation status yields a potential tool to assess chemosensitivity [4–7], but its role in clinical decision making remains controversial [4, 8–10]. Apart from that, present clinical imaging methods do not yield non-invasive biomarkers of response yet.

Chemical exchange saturation transfer (CEST) MRI might have the potential to fill this gap. CEST MRI is based on magnetic saturation of exchangeable protons in solute metabolites such as proteins, amino acids and lipids which serve as endogenous contrast agents [11–14]. A protein-weighted CEST-spectrum consists of distinct signals, mainly originating from the nuclear Overhauser effect (NOE) [13–15] and the amide proton transfer (APT) [12, 16]. Many CEST studies primarily extract APT signals using the asymmetric magnetic transfer ratio (MTR_asym_) [12, 17–20], which faces limitations due to overlap of different CEST signals and contributions of non-CEST effects [14, 21, 22]. Ultra-high magnetic fields enable high resolution CEST spectra, which in turn support a multi Lorentzian fit analysis to isolate NOE and APT signals [14, 22]. CEST effects are sensitive to biochemical [12, 14, 21, 23–25] as well as histopathological [19, 20, 26–28] tumor properties, therefore yielding complementary information to current MRI methods [12, 14, 22, 29]. Recently, even an accurate discrimination of distinct treatment-related changes in glioblastoma could be observed [18, 30].

In this work, we obtained high resolution protein-weighted CEST spectra at a 7 Tesla MRI scanner in untreated glioblastoma patients. We hypothesized that the resulting CEST contrasts can serve as predictors of treatment response in newly-diagnosed glioblastoma patients.

## RESULTS

To summarize the study design, all patients underwent protein-weighted CEST imaging on a 7T MRI prior to first-line treatment. The resulting CEST spectra were fitted using a five-pool multi-Lorentzian analysis which yielded the Lorentzian difference (LD) of NOE (NOE-LD) and APT (APT-LD) contrasts [14, 21]. A NOE-weighted MTRasym contrast was calculated as well [15]. In addition, the apparent exchange-dependent relaxation rate (AREX) was applied to correct for non-CEST effects (spillover, T1- and T2-relaxation, semi-solid magnetization transfer) [22]. Thus, NOE-AREX and APT-AREX contrasts were available, too. Finally, the downfield-NOE-suppressed (dns) APT was calculated to yield a further isolated APT contrast [29]. After the end of treatment, all patient cases were classified as early progression or stable disease based on the updated RANO criteria [31]. Subsequently, each CEST contrast was compared between the two groups of early progressors and stable disease based on the RANO criteria.

**Figure 1:**
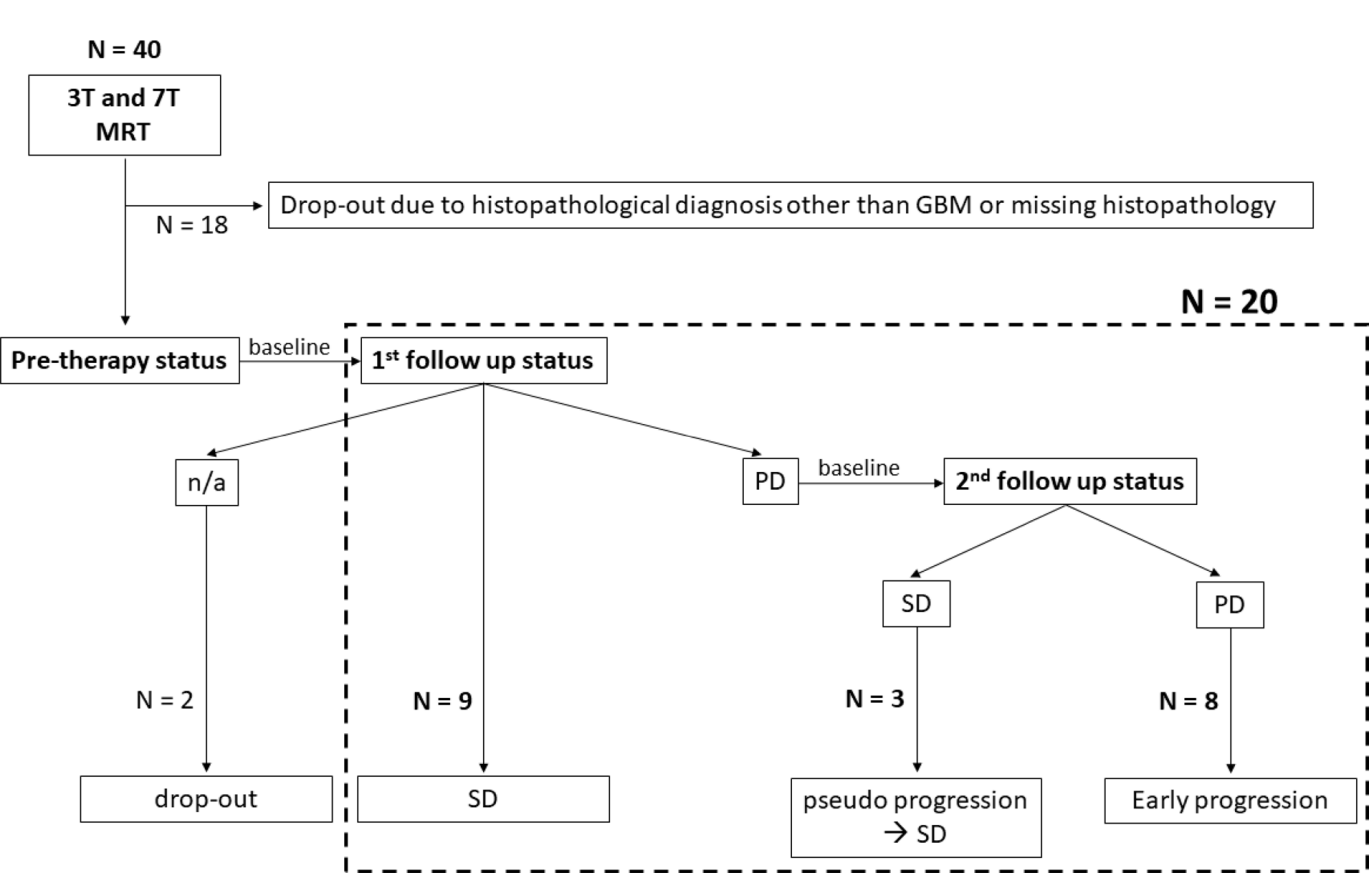
Enrollment of patients and consecutive response assessment (n/a = data not available, GBM = glioblastoma multiforme, SD = stable disease, PD = progressive disease).

Patient characteristics are summarized in Table 1. In the investigated study cohort, eight patients presented early progression following first-line standard treatment, whereas the disease remained stable in twelve patients (Figure 1).

**Table 1:** Patient characteristics

Characteristic				
**Age**	**[Years]**			
Median age	60			
Interquartile range	53–69			
**Sex**	***N***		**[%]**	
Male	12		60	
Female	8		40	
**IDH1-status**	***N***		**[%]**	
IDH1-mutation	1		5	
No IDH1-mutation	19		95	
**MGMT-promotor status**	**Stable disease (*N*)**	**Stable disease [%]**	**Early progression (*N*)**	**Early progression [%]**
methylated	4	33.3	3	37.5
Not methylated	4	33.3	5	62.5
Not assessed	4	33.3	0	0
**Surgery**	***N***		**[%]**	
Biopsy	8		40	
Subtotal resection	8		40	
Gross total resection	4		20	
**Surgery**	**Stable disease (*N*)**	**Stable disease [%]**	**Early progression (*N*)**	**Early progression [%]**
Resection	7	58.33	5	62.5
No resection	5	41.67	3	37.5
**Chemoradiation**	***N***		**[%]**	
60 Gy/2 Gy + TMZ	15		75	
40.05 Gy/2.67 Gy ± TMZ	5		25	
**Initial response (RANO)**	***N***		**[%]**	
Stable disease	12		60	
Early progression	8		40	

(IDH = isocitrate-dehydrogenase 1, MGMT = O6-methylguanine DNA methyltransferase, Gy = Gray, TMZ = temozolomide).

**Figure 2:**
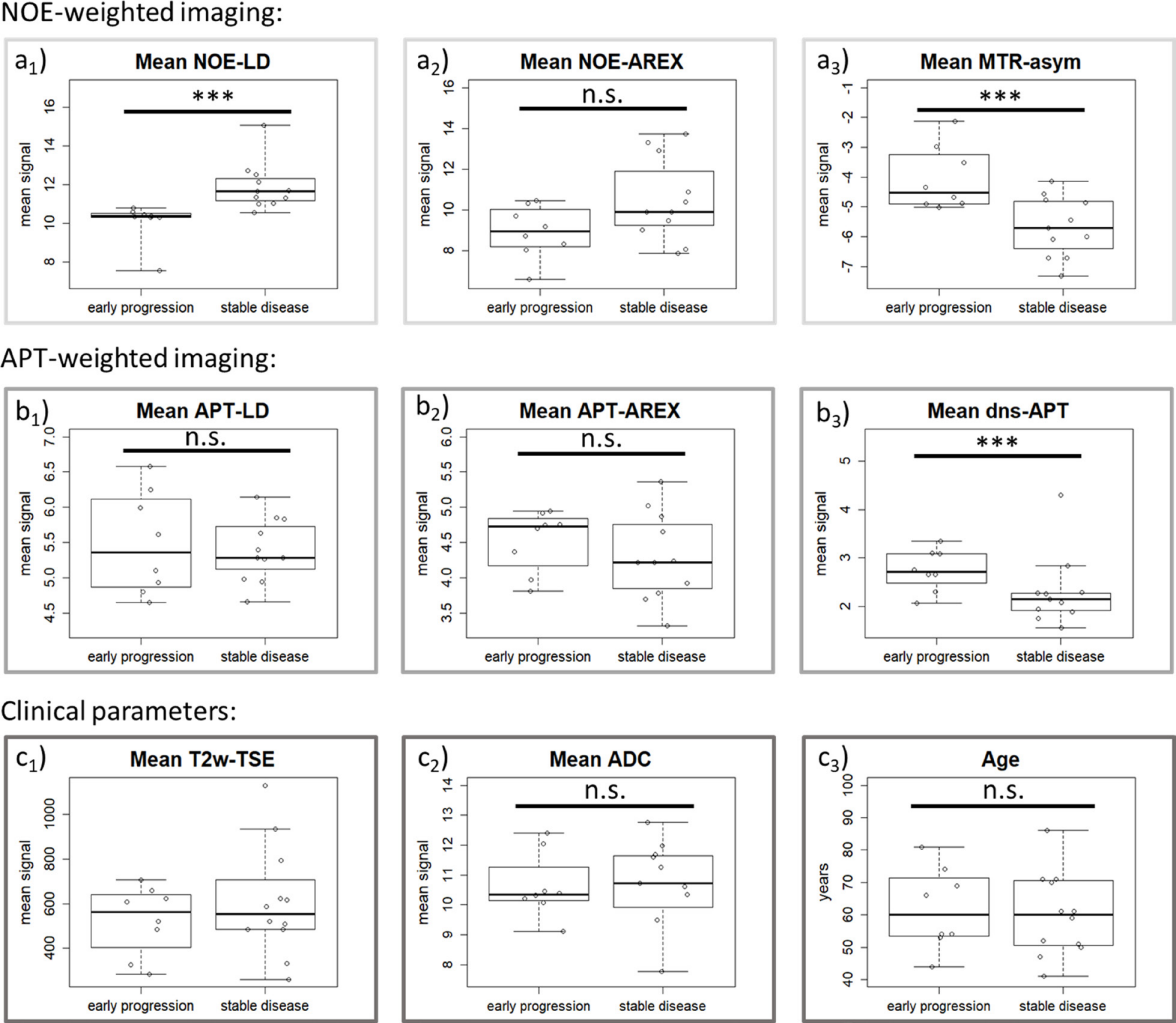
Description of pre-treatment differences for all parameters Pre-treatment differences of tumor mean signal intensity for all MRI contrasts as well as patient age regarding therapy response. NOE-weighted imaging: NOE-LD (**a**_**1**_) and -AREX (**a**_**2**_) show a lower signal in the patients with early progresses, whereas the opposite holds true for NOE-weighted MTR_asym_ (**a**_**3**_). APT-weighted imaging: APT-weighted contrasts show an increasing tendency towards higher values in the early-progressive group from left (APT-LD, **b**_**1**_) to right (dns-APT, **b**_**3**_). It seems that the more isolated the APT-contribution, the clearer this tendency gets. Clinical parameters: No clear intergroup difference in mean signal intensities of T2w-TSE at 7T (**c**_**1**_) and patient age can (**c**_**3**_) be observed. There seems to be a slight trend to lower values in early progression concerning mean ADC signals (**c**_**2**_). (^***^ = statistically significant according to Mann–Whitney *U* test with α ≤ 0.05, n.s. = not statistically significant).

**Figure 3:**
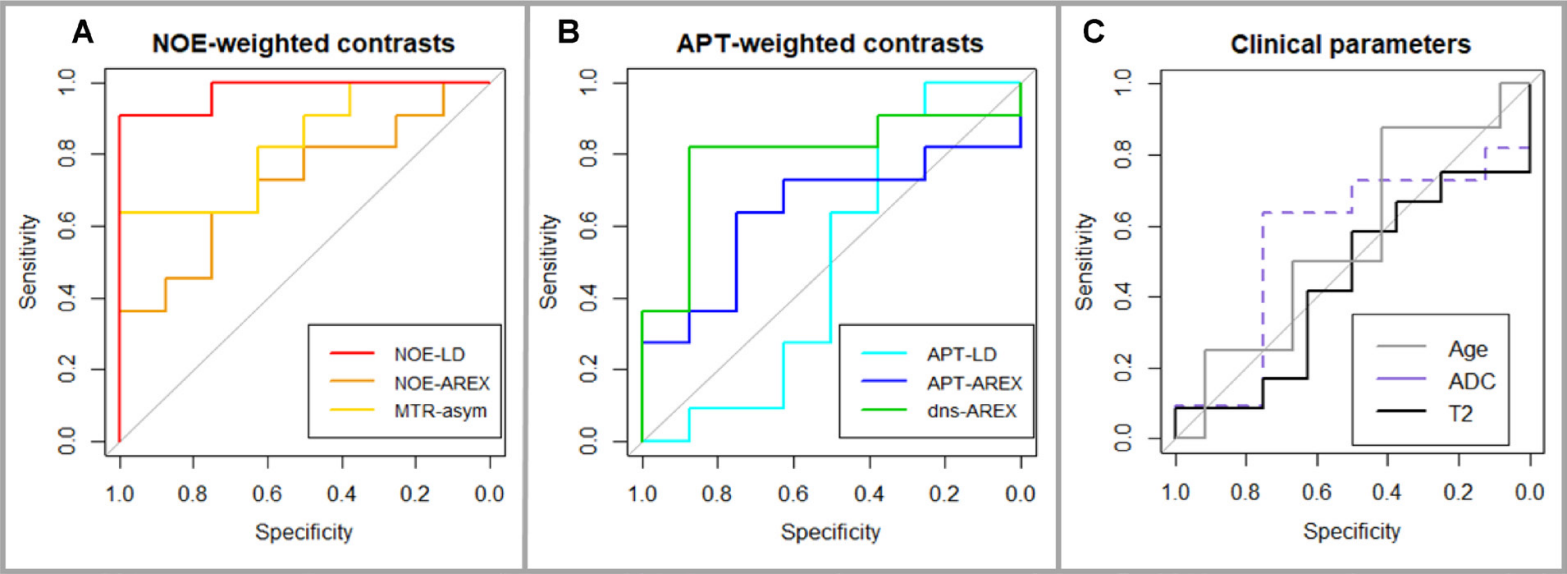
Receiver Operating Characteristics (ROC) graphs (**A**) NOE-weighted CEST including NOE-LD (red, AUC = 0.98, *p* = 0.0005) and MTR_asym_ (yellow, AUC = 0.83, *p* = 0.0166) which provide accurate prediction of early progression and reached statistical significance as opposed to NOE-AREX (orange, AUC = 0.72, *p* = 0.1167). (**B**) APT-weighted CEST with dns-APT (green, AUC = 0.80, *p* = 0.0318) being the only accurate and significant predictor of early progression compared to APT-AREX (blue, AUC = 0.64, *p* = 0.3218) and APT-LD (cyan, AUC = 0.50, *p* = 1). (**C**) Clinical parameters T2w-TSE at 7T (black, AUC = 0.56, *p* = 0.6434), ADC at 3T (dashed purple, AUC = 0.56, *p* = 0.6797) and patient age (grey, AUC = 0.56, *p* = 0.6434) do not show good predictive accuracy with AUC values close to 0.5 and did not reach statistical significance either.

Pre-treatment mean signal intensities of early-progressive tumors showed generally lower values on NOE-weighted contrasts (NOE-LD: median stable disease = 11.66, median early progression = 10.37, *p* = 0.0001 and NOE-AREX: median stable disease = 9.91, median early progression = 8.95, *p* = 0.1288). Accordingly, the NOE-weighted MTR_asym_ (median stable disease = *–*5.71, median early progression = *–*4.52, *p* = 0.0186) presented higher values in case of early progression (Figure 2, NOE-weighted imaging). The difference between early progression and stable disease reached statistical significance for NOE-LD and NOE-weighted MTR_asym_. For APT-weighted measures, there was a trend towards higher pre-treatment mean tumor signal intensities in early-progressive disease compared to stable disease (APT-LD: median stable disease = 5.28, median early progression = 5.36, *p* = 0.7725; APT-AREX: median stable disease = 4.22, median early progression = 4.73, *p* = 0.3218; dns-APT: median stable disease = 2.14, median early progression = 2.71, *p* = 0.0328) (Figure 2, APT-weighted imaging). This trend increased with isolation of the APT signal, reaching a significant difference between the two groups only in case of the most isolated dns-APT contrast. Correspondingly, ROC AUC analysis showed that a statistically significant prediction of early progression after first-line standard treatment was possible employing NOE-LD (AUC = 0.98, *p* = 0.0005), NOE-weighted MTR_asym_ (AUC = 0.83, *p* = 0.0166) and dns-APT (AUC = 0.80, *p* = 0.0318) measures (Figure 3A and 3B). The highest predictive accuracy was achieved utilizing the NOE-LD with a sensitivity of 91% and a specificity of 100%. MTR_asym_ yielded a sensitivity of 73% and specificity of 100%, whereas dns-APT reached sensitivity and specificity values of 82% and 88%.

None of the investigated clinical parameters (T2-weighted turbo spin echo sequence (T2w-TSE) at 7T: median stable disease = 554, median early progression = 563, *p* = 0.6784; apparent diffusion coefficient (ADC) at 3T: median stable disease = 10.72, median early progression = 10.35, *p* = 0.7168; Age: median stable disease = 60 years, median early progression = 60 years, *p* = 0.6710) presented any significant difference between early-progressive and stable disease, even though a slight tendency towards lower values in early progression was observed for the ADC contrast at 3T (Figure 2, Clinical parameters). Consequently, clinical parameters were not able to predict early progression according to ROC AUC analysis (Figure 3C). Furthermore, the descriptive analysis of MGMT-promotor methylation status and extent of surgery showed merely slight differences between patients experiencing early progression and stable disease for both parameters (Table 1). There was no significant interdependence of the MGMT-status and response to treatment (*p* = 1.00) or the extent of surgery and response to treatment (*p* = 1.00).

**Figure 4:**
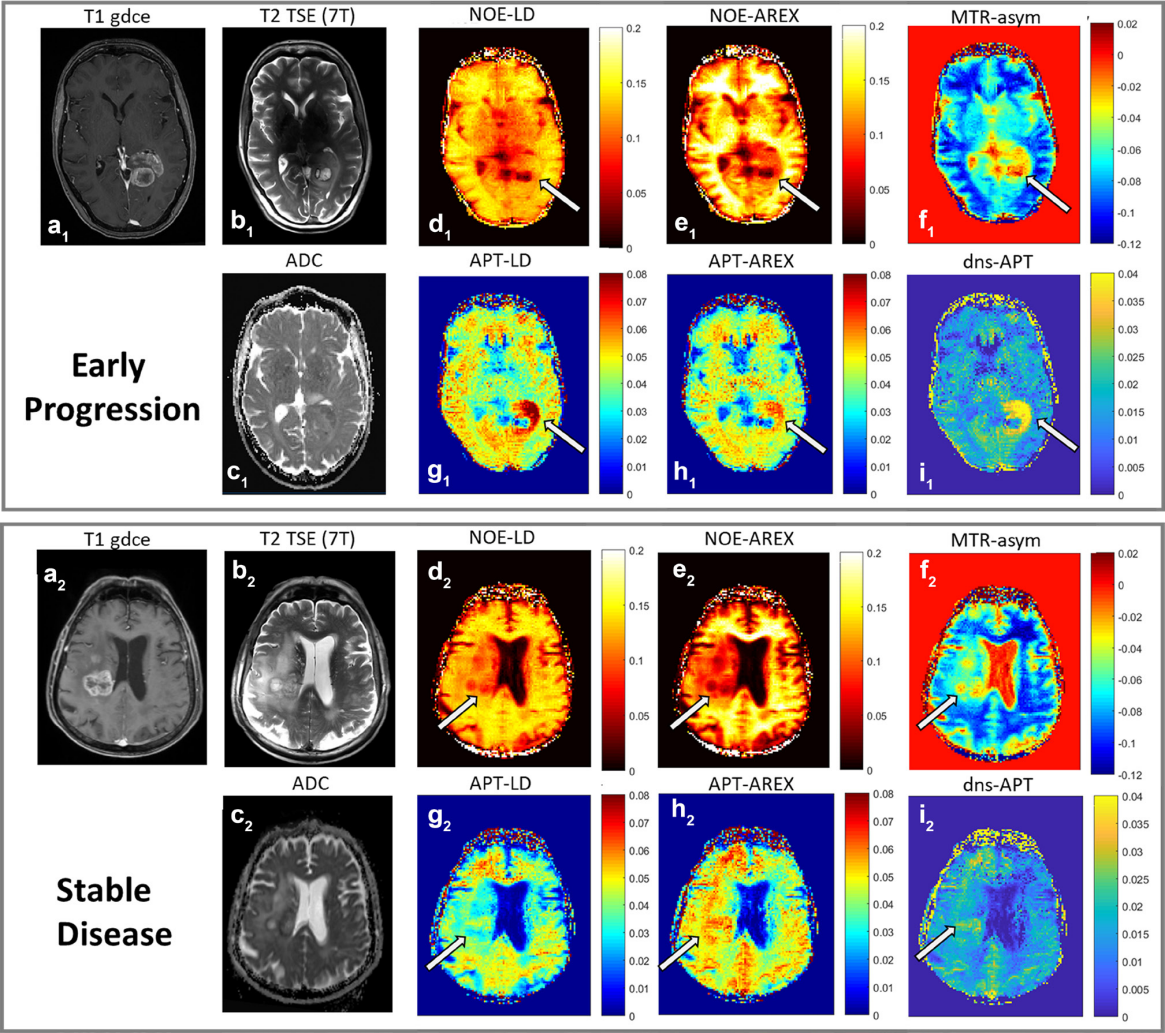
Exemplary CEST MR-images Top: Early progression. Bottom: Stable disease. **a**_**1**_ and **a**_**2**_: T1-weighting with gadolinium enhancement (3 Tesla), **b**_**1**_ and **b**_**2**_: T2-weighted TSE (7 Tesla), **c**_**1**_ and **c**_**2**_: ADC-map (3 Tesla), **d**_**1**_ and **d**_**2**_: NOE-LD, **e**_**1**_ and **e**_**2**_: NOE-AREX, **f**_**1**_ and **f**_**2**_: NOE-weighted MTR_asym_, **g**_**1**_ and **g**_**2**_: APT-LD, **h**_**1**_ and **h**_**2**_: APT-AREX, **i**_**1**_ and **i**_**2**_: dns-APT. NOE-LD and -AREX-mediated images show a decreased tumor signal, with much lower values in the early progression case (arrows in **d**_**1**_–**e**_**1**_ compared to **d**_**2**_–**e**_**2**_). The opposite is true for the MTR_asym_ contrasts (arrow in **f**_**1**_ compared to **f**_**2**_). APT-mediated images show a considerably higher signal intensity within the early-progressive tumor (arrows in **g**_**1**_–**i**_**1**_ compared to **g**_**2**_–**i**_**2**_).

All statistical findings are summarized in Table 2. Figure 4 shows exemplary patient images comparing an early progression with a stable disease for all investigated contrasts. Close-up views of tumor region are additionally provided (Supplementary Figure 1).

**Table 2:** Results of statistical analysis

Contrast	Stable disease (median & iqr)	Early progression (median & iqr)	*p*-val. (*U*-test)	AUC (95% CI)	*p*-val. (AUC)	Best cut-off	Sensitivity (95% CI)	Specificity (95% CI)
**NOE-LD**	**11.66** **(11.18–12.31)**	**10.37** **(10.31 – 10.48)**	**0.0001**	**0.98** **(0.92 – 1.00)**	**0.0005**	**10.89**	**0.91** **(0.82 – 1.00)**	**1.00** **(0.88 – 1.00)**
NOE-AREX	9.91 (9.25 – 11.90)	8.95 (8.25 – 9.87)	0.1288	0.72 (0.48 – 0.95)	0.1167	9.81	0.64 (0.27 – 1.00)	0.75 (0.38 – 1.00)
**MTR-asym (NOEw)**	**−5.71** **(−6.41 – − 4.82)**	**-4.52** **(**−**4.90 – 3.39)**	**0.0186**	**0.83** **(0.64 – 1.00)**	**0.0166**	−**5.23**	**0.73** **(0.45 – 1.00)**	**1.00** **(0.50 – 1.00)**
APT-LD	5.28 (5.12 – 5.39)	5.36 (4.90 – 6.06)	1.0000	0.50 (0.18 – 0.82)	1.0000	5.92	0.91 (0.18 – 1.00)	0.38 (0.13 – 1.00)
APT-AREX	4.22 (3.85 – 4.76)	4.73 (4.27 – 4.80)	0.3421	0.64 (0.37 – 0.90)	0.3218	4.3	0.64 (0.18 – 0.91)	0.75 (0.50 -1.00)
**dns-APT**	**2.14** **(1.92 – 2.28)**	**2.71** **(2.56 – 3.09)**	**0.0328**	**0.80** **(0.57 – 1.00)**	**0.0318**	**2.29**	**0.82** **(0.54 – 1.00)**	**0.88** **(0.63 – 1.00)**
T2w TSE	554 (485 – 666)	563 (444 – 632)	0.6784	0.56 (0.17 – 0.71)	0.6434	595	0.58 (0.33 – 0.83)	0.50 (0.13 – 0.88)
ADC (3 Tesla)	10.72 (9.92 – 11.63)	10.35 (10.17 – 10.86)	0.7168	0.56 (0.27 – 0.85)	0.6797	10.53	0.64 (0.09 – 0.91)	0.75 (0.38 – 1.00)
Age	60 years (51 – 70 years)	60 years (54 – 70 years)	0.6710	0.56 (0.29 – 0.83)	0.6434	52.5	0.88 (0.63 – 1.00)	0.42 (0.17 – 0.67)

(iqr = interquartile range, AUC = area under curve, CI = confidence interval).

## DISCUSSION

In this study, we investigated high resolution CEST spectra obtained on a 7T MRI scanner allowing for simultaneous quantification of isolated APT- and NOE-weighted contrasts in glioblastoma patients prior to treatment. We showed that NOE-mediated contrasts, namely the NOE-LD and NOE-weighted MTR_asym_, as well as the dns-APT contrast enabled accurate prediction of early progression following first-line treatment.

At present, early evaluation of therapy response in glioblastoma is a huge clinical challenge. The state of the art is based on repeated post-treatment MRI examinations which face major limitations early after treatment due to possible pseudo progression [31–33]. Thus, a reliable identification of patients who did not respond to adjuvant CRT is substantially delayed, which in turn results in delayed start of salvage therapies indicated in these cases. This is particularly problematic in view of the fact that many patients suffer from early tumor progression [1, 3]. Recently, CEST MRI already showed the potential to enhance post-treatment response evaluation in glioblastoma [18, 30]. A pre-treatment assessment of tumor sensitivity towards standard therapy would allow the assignment of patients to the most promising therapeutic approach right from the beginning. MGMT promotor methylation status seems to be a helpful tool to assess chemosensitivity especially in the elderly population [4–7]. However, its clinical value concerning younger glioblastoma patients remains controversial due to several methodological and normative limitations [4, 8–10]. At this point, broadening the application of CEST MRI in the diagnostic work-up to obtain pre-treatment response predictors might be highly valuable. The resulting non-invasive imaging biomarkers could provide a convenient way to repeatedly evaluate the tumor throughout disease management. Moreover, CEST MRI might guide biopsies to relevant tumor sites, thus also enhancing the efficacy of histopathological tools. This could be an important step towards individualized therapy.

To our knowledge, this is the first approach employing CEST MRI as predictor of glioblastoma response to standard therapy in previously untreated patients. So far, APT imaging has shown promising results in post-treatment evaluation of tumor response. A drop in APT-signal after chemotherapy [34] or radiotherapy [35] correlated with decreasing cellularity and might serve as biomarker of response in glioblastoma. Furthermore, APT-weighted imaging could significantly distinguish between tumor progression and treatment related changes in glioblastoma with high APT values being characteristic for progression [18, 30]. Consequently, APT signal seems to be correlated with proliferation [18, 20, 34], thus possibly reflecting tumor aggressiveness and response to therapy. This is in accordance with higher pre-treatment APT signal values in early-progressing tumors found in our work. However, only the most isolated APT-mediated contrast showed a statistically significant difference regarding response to therapy. This result is in agreement with a recent study by Zhang *et al.* reporting increased correlation with tumor proliferation for isolated APT signals compared to conventional approaches based on MTR_asym_ [36].

Concerning NOE-mediated contrasts, it was previously shown that post-treatment NOE-weighted signals can significantly differentiate between tumor progression and radiation necrosis in brain metastases [37] whereas no significant correlation of any pre-treatment CEST signals in tumor tissue with consecutive therapy response could be found [38]. This is partly contrary to our study results, where NOE-weighted CEST provided highest diagnostic accuracy. However, it is important to note that Desmond *et al.* [38] worked with different imaging parameters because the particularly low B_1_-amplitudes (0.6–1.0 µT) which we applied at a static field of 7T especially enhance NOE-mediated CEST effects [14, 15, 23, 28, 39]. In addition, we investigated glioblastoma patients instead of brain metastasis, with numerous studies describing a drop of NOE signals within glioma [14, 15, 22, 28, 29]. This drop seems to be more pronounced in higher-grade tumors [40], which corresponds with our finding of predominantly low NOE values in early-progressing tumors. Better isolation of different CEST signals also led to a better predictive accuracy when comparing the NOE-LD with the NOE-weighted MTR_asym_. However, the additionally relaxation-compensated NOE-AREX metric yielded lower AUC values than NOE-LD. This is most likely due to a higher standard-deviation of NOE-AREX values resulting from the inverse metric approach [21]. The higher statistical errors of NOE-AREX values may in turn have decreased the effect size in our patient sample.

The origins of the different CEST signals are subject to ongoing research, but previously demonstrated contributions of protein concentration [14, 25], pH [12, 21, 25], protein conformation [23, 24, 41] and cellular proliferation [18, 20, 28, 34] imply that CEST MRI might yield non-invasive biomarkers for essential tumor characteristics.

This study found an essential difference in NOE- and APT-weighted CEST contrasts as predictors of early progression in glioblastomas. NOE-weighted contrasts showed a decrease in early-progressive tumors, whereas APT-weighted measures showed an opposite trend with increased APT signals in early progressors. As mentioned above, both APT and NOE mediated signals are primarily associated with protein/ peptide content and cellularity. Since APT and NOE show opposite signal alteration in tumor tissue, this result cannot only be caused by variations in protein concentration. A possible explanation is the decomposition of proteins/ peptides in tumors yielding an increased proportion of smaller protein fragments and peptides [42]. This would, in turn, result in increased APT effects (better accessibility of amide protons to bulk water exchange) and simultaneously decreased NOE signal, since the NOE has been shown to be strongly affected by protein size and conformation [23, 24, 41]. These effects were even more pronounced in the group of early progressors, possibly reflecting particularly aggressive tumor tissue.

A frequently reported pH-dependence of NOE effects, with low values being associated to low pH, has most likely only minor contributions to the observed signal change [14].

Moreover, CEST contrasts have been shown to be sensitive to histopathological features of gliomas such as tumor grade, isocitrate-dehydrogenase (IDH) -mutations and MGMT promotor status [19, 20, 26, 27, 43]. This supports the approach of employing CEST MRI as early response predictor in glioblastoma. Furthermore, all evaluated CEST signals provide complementary information to clinical MRI and methodological isolation clearly separates different CEST effects from each other [21, 28, 29]. Consequently, future approaches should investigate multiparametric prediction models based on various isolated CEST contrasts, traditional MRI parameters, and clinical information in order to further increase diagnostic performance of MRI.

Our study has some limitations. First, validation in a bigger patient trial is mandatory. However, this is the first study examining the value of multi-pool CEST MRI as response predictor, yielding statistically significant results in a prospective setting. Secondly, 7T MRI scanners are only available at few centers today which limits immediate implementation. Nevertheless, successful isolation of CEST-effects at clinical MRI scanners (3.0 T) in various diagnostic studies [36–38, 44] showed that translation of our findings into standard protocols is feasible. Still, our results are only valid for low B1-amplitudes at the high B0-field of 7T and reproducibility at clinical scanners needs future verification. Another possible limitation is the assessment of general standard first-line therapy involving different surgical approaches and age-adapted radiotherapy protocols in elderly glioblastoma patients. However, our study cohort represents a common clinical spectrum, which seemingly did not impede the significance of CEST MRI, underlining the robustness of this method. Furthermore, we did not find a significant difference between early progression and stable disease according to patient age or performed surgical approach. In contrast, CEST MRI yielded significant intergroup differences, thus providing the only considerable predictors of early tumor progression in this trial. Finally, the correlation of CEST effects with prognostic histopathological features [19, 20, 26, 27, 36] could have interfered with response prediction. However, we exclusively included grade IV glioblastomas and just one patient carried an IDH1-mutation in this study. Moreover, our findings showed only slight differences in MGMT-promotor status regarding therapy response. This strongly suggests that CEST based contrasts, which yielded significant prediction of early tumor progression, provide complementary information to histopathology. Ultimately, the response assessment serving as a reference in this study is based on the updated RANO criteria [31], which face limitations in the initial setting due to possible pseudo progression [32, 33]. Nevertheless, RANO criteria are increasingly employed in neuro-oncology trials as the current state of the art in objective response assessment. In our study, we followed the recommendations of the RANO working group to validate apparent tumor progression within 12 weeks post-treatment through a second follow-up examination [31]. This allowed us to detect early pseudo progression successfully in several patients.

In conclusion, multi-pool CEST MRI derived contrasts, particularly NOE-weighted imaging, yielded the potential to predict early tumor progression after first-line therapy in previously untreated glioblastoma patients and might therefore be a promising non-invasive tool for customization of treatment in the future.

## MATERIALS AND METHODS

### Patients

From October 2015 to September 2017, 40 consecutive patients with recently-diagnosed intracranial tumors underwent a 7T MRI examination prior to treatment. MRI inclusion criteria were an age of 18 years or older, prior findings suspicious for glioma, no previous treatment and eligibility for 7T MRI. Moreover, for evaluation of the predictive capacities of CEST at 7T, histopathologically confirmed diagnosis of glioblastoma multiforme (GBM, WHO grade IV) and complete clinical records from follow-up examinations were required. Finally, 20 patients were enrolled in this study, whereas the rest had to be excluded due to histopathological diagnosis other than glioblastoma or missing clinical data (Figure 1). Eleven patients were previously reported as part of a different patient cohort [22, 29]. The CEST spectra obtained in one patient were strongly distorted due to motion artifacts, hence only clinical imaging of this patient was eligible for evaluation. Patient characteristics are summarized in Table 1. Resection was performed in twelve patients, whereas eight did not receive a therapeutic resection. Subsequently, all patients underwent adjuvant treatment consisting of radiotherapy (standard protocol: 60 Gray, 30 fractions) with concomitant (75 mg/m^2^) and adjuvant (150–200 mg/m^2^) administration of temozolomide. Therapy was adapted following suggestions for elderly [6, 7] in five patients (40.05 Gy, 15 fractions and/or temozolomide in the standard dose).

### Response assessment

Patient response status to treatment was determined based on clinical 3T MRI and neurological evaluation derived from the first and second follow-up examinations (approx. one and three months after the end of radiotherapy). Radiological findings were routinely evaluated by the department of neuroradiology in accordance with the updated RANO criteria [31]. Subsequently, comprehensive evaluation of radiological and neurological status was done by a neurooncologist following the updated RANO criteria [31]. Results obtained in each examination were rated as complete response (CR), partial response (PR), stable disease (SD) or progressive disease (PD). For all patients classified as potential PD in the first follow up, the rating of the second follow up was considerable to account for possible pseudo progression within the first 12 weeks [31–33]. Only if evaluation of both time steps led to the conclusion that PD was present, this case was rated as PD in accordance with the RANO criteria [31]. Otherwise, the classification of the second follow-up overruled the initial rating, thus defining a pseudo progression.

The whole workflow of this study including the response assessment is summarized in Figure 1. In total, twelve cases were classified as SD. Conversely, eight patients suffered from PD, thus early progression was present.

### Clinical MRI at 3T

Clinical MRI exams were performed at 3T prior to therapy and as part of follow up examinations based on the following protocol parameters: T2w-TSE [echo time (TE) = 86 ms; repetition time (TR) = 5550 ms; field of view (FoV): 229 × 172 mm^2^; matrix: 384 × 230; slice thickness: 5 mm], T2-weighted fluid attenuated inversion recovery (T2w-FLAIR) [TE = 135 ms; TR = 8500 ms; FoV: 230 × 172 mm^2^; matrix: 256 × 192; slice thickness: 5 mm], T1-weighted gadolinium contrast-enhanced (T1w-gdce) [TE = 4.04 ms; TR = 1710 ms; FoV: 256 × 256 mm^2^; matrix: 512 × 512; slice thickness: 1 mm] and diffusion imaging yielding the ADC [echo planar readout, TE = 90 ms; TR = 5300 ms; b = 0 s/mm^2^ and b = 1200 s/mm^2^; FoV: 229 × 229 mm^2^; matrix: 130 × 130; slice thickness: 5 mm]. This is in accordance with the consensus recommendations for standardized brain tumor imaging and response evaluation as proposed by Ellingson *et al.* [45].

### CEST MRI at 7T

The examinations were performed on a 7T MRI scanner (MAGNETOM 7.0 Tesla; Siemens Healthcare, Erlangen, Germany) with a single channel transmit/24 channel receive ^1^H head coil (Nova Medical, Wilmington, USA) prior to therapy. CEST imaging based on a custom-developed 2D gradient echo (GRE) sequence according to Zaiss *et al.* [22]. In detail, images were obtained after saturation [train of 150 Gaussian shaped radiofrequency (RF) pulses, t_p_ = 15 ms, t_d_ = 10 ms, duty-cycle = 60%, t_sat_ = 3.75 s] for two distinct B_1_ amplitudes [1.0 μT and 0.6 μT] and for three adjacent slices [slice thickness = 5 mm] [22]. MTR_asym_ was calculated at 3.5 ppm [16], yet yielding a predominantly NOE-mediated contrast due to the low B_1_-amplitudes (0.6–1.0 µT) applied at 7 Tesla [14, 23, 39]. Separation of NOE and APT-mediated CEST effects was based on a multi-Lorentzian fitting approach as previously described [22], which was applied on the high-resolution CEST spectra at 7T. Subsequently, different NOE- and APT-weighted contrasts were evaluated at B_1_ = 0.6 µT and corrected for field inhomogeneities according to Windschuh *et al.* [46], the first being the Lorentzian difference (LD) [14]. Additional application of the relaxation-compensated metric according to Zaiss *et al.* [21] led to the apparent exchange-dependent relaxation rate (AREX) which is corrected for spillover, T1- and T2-relaxation and semi-solid magnetization transfer [22]. Furthermore, residual overlap of downfield-resonating NOE with the APT signal was removed using the downfield-NOE-suppressed (dns) APT contrast as reported by Zaiss *et al.* [29]. Mapping of B_0_ and B_1_ inhomogeneities was achieved following the simultaneous mapping of water shift and B_1_ (WASABI) approach [47]. In total, the CEST MRI scans required 22–25 min including shimming, B_0_/B_1_ correction and relaxation-compensation. Exemplary CEST spectra from the tumor region and contralateral normal appearing white matter are provided, both for a patient with early progression and stable disease (Supplementary Figure 2).

In addition, high-resolution T2w-TSE imaging was performed [TE = 54 ms; TR = 14130 ms; FoV: 220 × 178.8 mm^2^; matrix: 512 × 416.3; slice thickness: 2 mm] as previously reported [48].

### Data analysis

Co-registration of all images was performed employing an automatic multi-modal rigid registration algorithm in MITK [49]. Manual segmentation of the tumor region including all areas of abnormal signal intensity on T1-gdce and T2-weighted images but excluding necrosis was done by an experienced neuroradiologist (A.R., 10 years of experience). This approach enables better reproducibility than manual selection of distinct regions of interest within the tumor. Subsequently, the mean signal intensities over all included voxels were calculated for the ADC obtained at 3T and for all multi-pool CEST contrasts and the T2w-TSE obtained at 7T.

### Statistical analysis

The median and interquartile range of the tumor mean signal intensities were calculated for each contrast as well as patient age in the early-progression and stable disease group respectively. Mann–Whitney *U* tests were performed to compare the signal intensities and age between patients with stable disease and patients experiencing early progression. In addition, receiver operating characteristic (ROC) area under the curve (AUC) analyses were applied to assess prediction of early progression. Consecutively, best thresholds were determined for each contrast following Youden’s J statistics. Moreover, a possible interdependence of the MGMT-promotor status and therapy response as well as the extent of the surgical approach and therapy response was tested employing Fisher’s exact test. The data analysis employed R version 3.4.3 and the pROC package [50]. The level of significance was set to *p* < 0.05 for all performed tests.

### Ethics statement

This study was conducted in accordance with the declaration of Helsinki and received approval by the local ethics committee. MRI examinations were performed after written informed consent was obtained from the patient.

## SUPPLEMENTARY MATERIALS FIGURES



## Abbreviations

ADC: apparent diffusion coefficient
APT: amide proton transfer
AREX: apparent exchange-dependent relaxation rate
AUC: area under the curve
CEST: chemical exchange saturation transfer
CR: complete response
CRT: chemoradiotherapy
dns-APT: downfield- nuclear Overhauser effect -suppressed amide proton transfer
FLAIR: fluid attenuated inversion recovery
FoV: field of view
GBM: glioblastoma multiforme
gdce: gadolinium contrast-enhanced
GRE: gradient echo
IDH1: isocitrate-dehydrogenase 1
LD: Lorentzian difference
MGMT: O6-methylguanine DNA methyltransferase
MITK: medical imaging interaction toolkit
MRI: magnetic resonance imagin
MTR_asym_: asymmetric magnetic transfer ratio
NOE: nuclear Overhauser effect
PD: progressive disease
PR: partial response
RANO: response assessment in neuro-oncology working group
RF: radiofrequency
ROC: receiver operating characteristic
SD: stable disease
T: Tesla
TE: echo time
TMZ: Temozolomide
TR: repetition time
TSE: turbo spin echo
WHO: world health organization
