# Supplementary material for: Chemical exchange saturation transfer MRI serves as predictor of early progression in glioblastoma patients
Source: Oncotarget. 2018 Jun 19;9(47):28772–83. doi: 10.18632/oncotarget.25594 (PMC6033360; doi:10.18632/oncotarget.25594)
Supplement: Supplementary file 1 [file oncotarget-09-28772-s001.pdf]

Chemical exchange saturation transfer MRI serves as predictor of early progression in glioblastoma patients

SUPPLEMENTARY MATERIALS

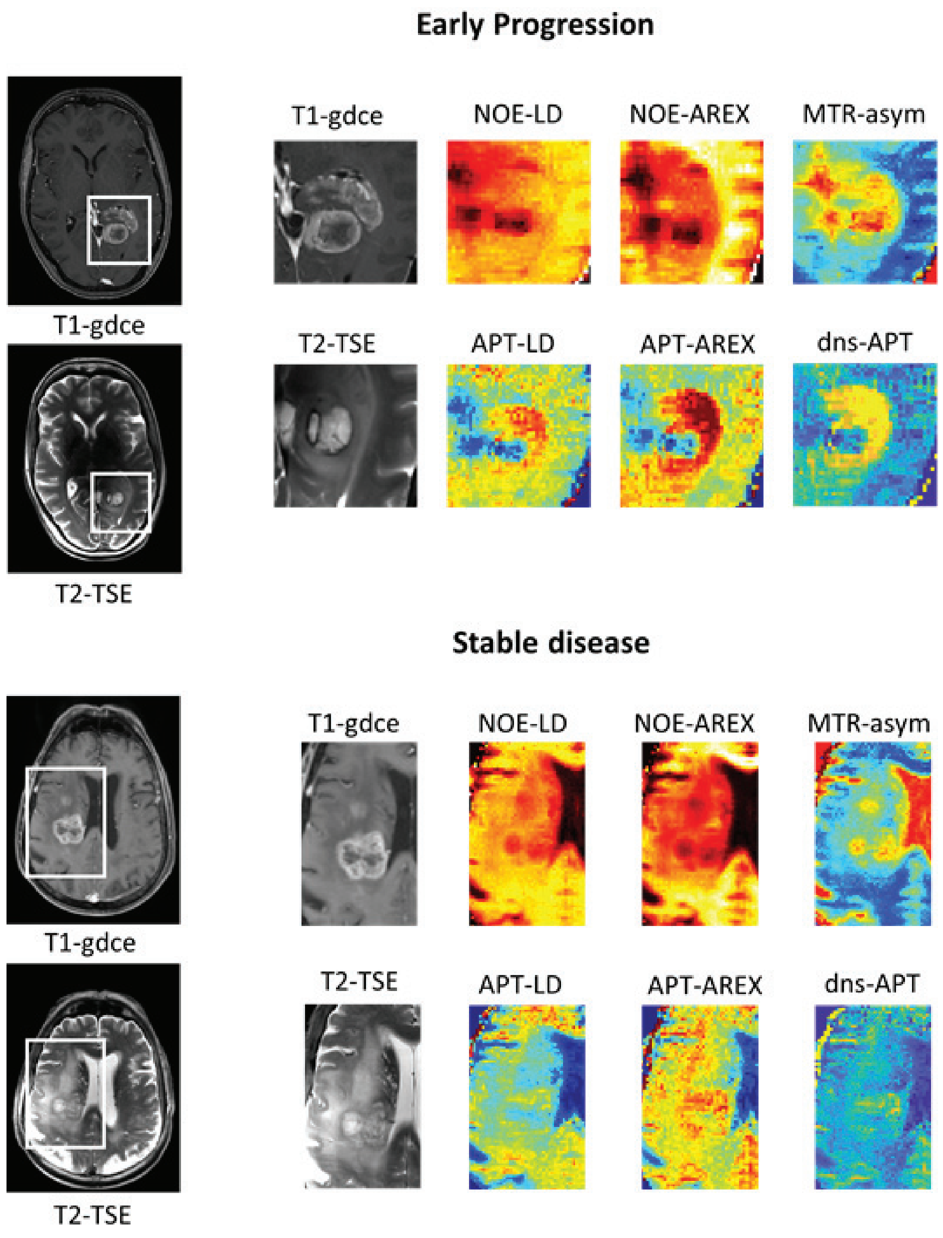

Supplementary Figure 1: Close-up views of the tumor regions in one patient with early-progressive and another patient with stable disease. The magnified tumor region is delineated on clinical 3T MRI with a white frame.

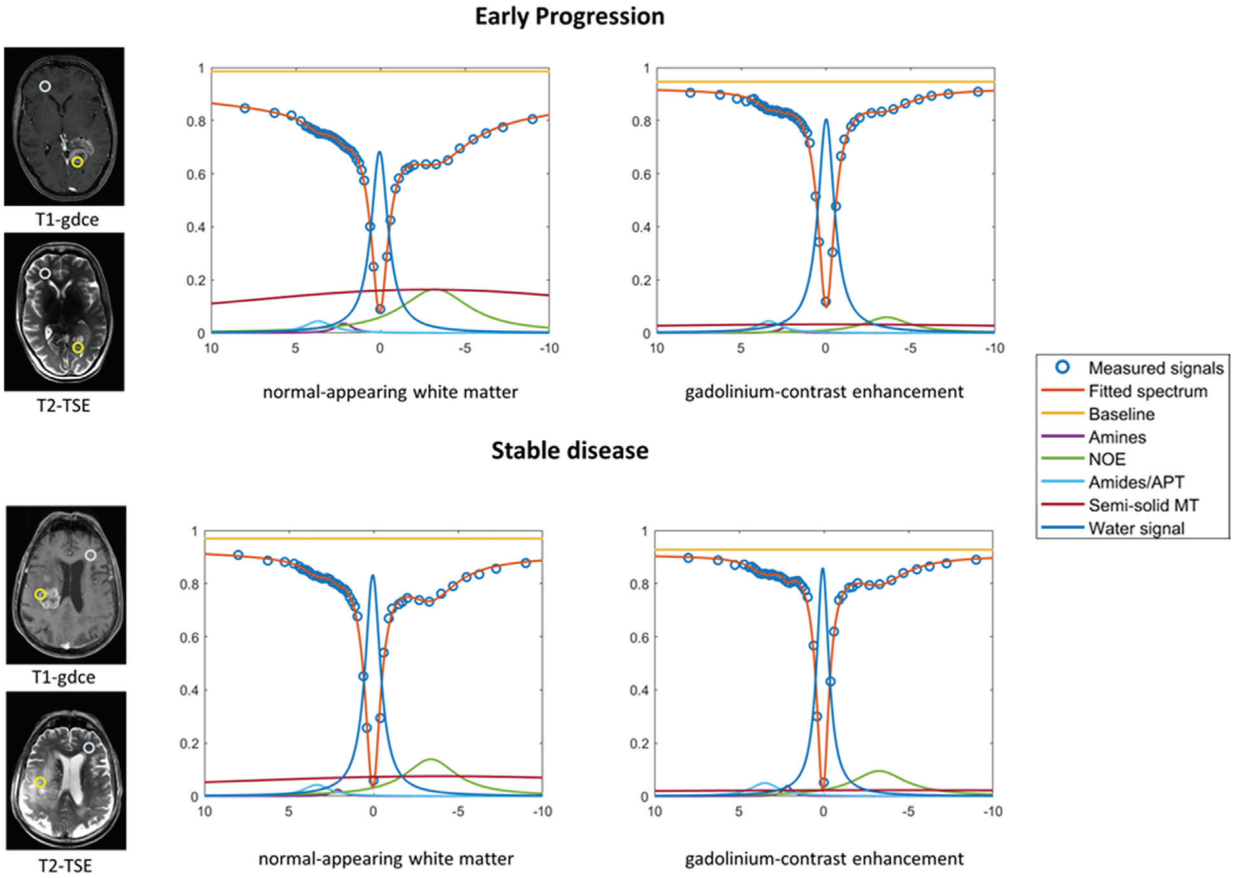

Supplementary Figure 2: CEST-spectra in one patient with early-progressive and another patient with stable disease. On the left side, T1-weighted gadolinium-enhanced (T1-gdce) and T2-weighted turbo-spin-echo (T2-TSE) MRI are shown together with the regions of normal-appearing white matter (light-blue circles) and gadolinium-contrast enhancement (yellow circles) where exemplary CEST-spectra were measured. It should be noted that the corresponding CEST-spectra represent only one single CEST-MRI voxel and not the whole region. The actually discontinuous spectrum is depicted as blue circles which correspond to the signals measured at the different CEST offset frequencies. Those data points were fitted to yield a continuous spectrum (orange line). Subsequently, a decomposition of the spectrum into five different Lorentzian functions isolated the distinct CEST effects (dark blue: bulk water signal, dark red: semi-solid MT, purple: Amines, light blue: Amides/APT, green: NOE). When comparing the tumor spectra (right) with those of normal-appearing white matter (left), the increased APT as well as decreased NOE signals in the tumor area can be observed. However, both changes are more pronounced in the early-progressive tumor.
